# Rheological and textural properties of lafun, a stiff dough, from improved cassava varieties

**DOI:** 10.1111/ijfs.14902

**Published:** 2020-11-28

**Authors:** Alexandre Bouniol, Laurent Adinsi, Sègla Wilfrid Padonou, Francis Hotegni, Désiré Gnanvossou, Thierry Tran, Dominique Dufour, Djidjoho Joseph Hounhouigan, Noël Akissoé

**Affiliations:** ^1^ Laboratoire de Sciences des Aliments Faculté des Sciences Agronomiques Université d’Abomey‐Calavi Jéricho 03 BP 2819 Benin; ^2^ CIRAD UMR QUALISUD Cotonou 01 BP 52 Benin; ^3^ Qualisud Univ Montpellier CIRAD Montpellier SupAgro Univ d’Avignon Univ de La Réunion 73 avenue JF Breton, Montpellier Cedex 5 Montpellier 34398 France; ^4^ ESTCTPA Université Nationale d’Agriculture Porto‐Novo 01 BP 55 Bénin; ^5^ International Institute of Tropical Agriculture (IITA) 08 BP 0932 Tri Postal Cotonou Bénin; ^6^ The Alliance of Bioversity International and the International Center for Tropical Agriculture (CIAT) CGIAR Research Program on Roots Tubers and Bananas (RTB) Apartado Aéreo 6713 Cali Colombia; ^7^ CIRAD UMR QUALISUD Montpellier F‐34398 France

**Keywords:** Cassava roots, hardness, pasting properties, provitamin A carotenoids, stickiness, viscoelastic

## Abstract

We studied the textural and rheological (viscoelastic) properties of fresh lafun dough, a fermented cassava product, and their changes during storage at 45 °C for 5 and 24 h, in order to determine after‐cooking storability. Lafun flours were produced from three types of cassava varieties: seven improved white‐fleshed varieties, seven improved provitamin A carotenoids (pVAC) varieties and two local white‐fleshed varieties; and processed into lafun doughs. Pasting properties of the flours were assessed. Flours from local varieties had pasting profiles with highest viscosities, while pVAC flours had the lowest. The three types of cassava varieties varied significantly in most of their pasting properties. Four promising improved varieties were identified, based on high peak viscosity (55.8–61.5 P) and stiffer texture than local varieties during storage. Undesirable varieties were also found, which softened during storage instead of hardening. Optimum texture of lafun dough was obtained after 5 h of storage.

## Graphical abstract

Fermented cassava dough (lafun) showed three textural behaviours during storage: stiffening, softening and no modification. Promising improved varieties had hardness behaviour close to local varieties and optimum texture of this dough is around 5 h.

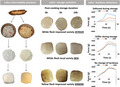

## Introduction

Cassava (*Manihot esculenta* Crantz) is cultivated in tropical and subtropical regions for its starchy roots, whose flesh is white, or less commonly cream to yellow. Increasing interest is oriented to the cassava plant as this crop is more and more used for food and nutritional security purposes and for many industrial applications. It constitutes a staple food for more than 500 million people (El Sharkawy, 2004) and is consumed in various food forms, including traditional and newly designed foods. To meet the requirement of the users, improved varieties with particular properties (fast growth, high yield, low cyanogenic potential, provitamin A biofortified, etc.) have been developed (Teeken *et al*., 2018). Particularly, as vitamin A deficiency is a widespread public health concern in sub‐Saharan Africa, breeders released provitamin A biofortified cassava to enhance this micronutrient intake. As far as micronutrient deficiency in white‐fleshed cassava varieties is concerned, cassava consumers can improve their diet with provitamin A enriched derived foods such as gari, fufu, lafun, chickwangue and attiéké. However, apart from nutritional qualities, consumers expect specific textural characteristics from these foods (firmness, stickiness, chewiness, adhesiveness, extensibility, swelling capacity, etc.), which are often not well known for new genotypes.

Several studies have been carried out on pasting and rheological properties of flour and starch derived from cassava. Ojewumi *et al*. (2019) studied the rheological properties of cassava starch in comparison with corn starch and reported that cassava starch gel had a more pseudo‐plastic property. In Ghana, Aryee *et al*. (2006) evaluated thirty‐one cassava varieties for their pasting characteristics. In Nigeria, Omodamiro *et al*. (2007) processed twenty‐eight cassava genotypes into starch and lafun and measured pasting and functional properties. Anggraini *et al*. (2009) reported that white‐fleshed and yellow‐fleshed cassava were similar in elasticity, cohesiveness, adhesiveness and hardness of the starch gel. However, Awoyale *et al*. (2015) found pasting temperature, peak viscosity, breakdown, setback and final viscosities to be higher for yellow‐ than for white‐fleshed cassava varieties. Other properties such as water absorption capacity, swelling power and solubility were shown to be location‐ and variety‐dependent on both yellow‐ and white‐fleshed cassava (Anggraini *et al*., 2009; Awoyale *et al*., 2015; Ayetigbo *et al*., 2018). However, for Ayetigbo *et al*. (2018), flours from both types of cassava displayed similar textural and rheological properties. Although the white‐ and yellow‐fleshed cassava varieties have been studied regarding properties of their starches and flours, limited research has been done on the relationship between flour pasting properties and dough texture, and the effect of storage conditions on cassava dough like lafun.

Lafun is made by fermenting and drying cassava chunks, which are then milled into flour. To obtain a stiff dough, lafun flour is added to a mixture of boiled water and ogi, a maize starch. Nago *et al*. (1998) reported starch content in ogi varied from 75% to 77%, while in fermented cassava, it varied from 77% to 85% (Onitilo *et al*., 2007). Because of the high pasting characteristics of cassava starch (Anggraini *et al*., 2009; Ojewumi *et al*., 2019), processors add the ogi during lafun cooking to decrease viscosity and thereby reduce the strength required for kneading the dough. This behaviour was highlighted by Monthe *et al*. (2019) who reported that cassava flour had high pasting characteristics (due to high starch and low amylose contents), leading the authors to add cereal flour in composite bread preparation. These authors reported that hardness of the derived product seems to be positively correlated with the sorghum proportion and negatively with the cassava proportion.

The fermentation process can impact stability of starch granules (Adegunwa *et al*., 2012). The addition of ogi (maize starch) presumably affects after‐cooking stability, and as a result, lafun could show particular texture and rheological properties. Other sources of variability include the fact that after cooking, the dough can be eaten or stored for one to three days, sometimes with a loss of textural quality. There is a lack of information on the texture of lafun dough during storage. This study aims at clarifying textural and rheological properties, and after‐cooking storability of lafun from improved cassava varieties, and positioning them against lafun from two control local varieties.

## Materials and methods

### Raw material

Two local cassava varieties and fourteen improved varieties were studied, all of them with low cyanogenic potential (*sweet*). Local varieties [(EYEKOFO and Ben‐86052 (Ben)] have white flesh, and they were selected based on the recognised suitability for lafun production by processors. All the improved varieties were from an experimental plot established for morphological characterisation at the IITA‐Benin station during the 2017–2018 cropping season. Among these cassava varieties, seven (I011797, I083724, I011412, I083594, I070593, I090090, I070539) were provitamin A carotenoid (pVAC) with a yellow flesh, and seven [Ina‐H (92B/00061), Igbèkokpan (92B/00068), MR‐67 (92/0067), RB 89509 (RB or Gbézé), Ina Premier (92/0427), Maniben‐03 (Oko‐Iyawo), Obaïlè (92B/0057)] had white flesh. Roots were harvested 12 months after planting.

Ogi was produced as described by Nago *et al*. (1998) by boiling whole maize kernels for 10 min, steeping for 1 h, wet‐milling and wet‐sieving using tap water in the ratio (water: ground product) of 5:1. After settling for 12 h, the supernatant was poured off until 28–30% dry matter content to obtain ogi.

### Cassava processing into lafun flour

The sixteen experimental materials were processed separately by a consortium of skilled processors. Cassava roots (35 kg) were peeled, roughly cut and washed. Cut pieces (7–10 cm length) were soaked in tap water, with each material contained in separate covered plastic containers (100 L), and left to ferment for 48 h at ambient temperature (28–30 °C). Thereafter, the fermented cassava pieces were defibred and crumbled by hand, placed on trays and then sun‐dried for three days. Samples were covered overnight and uncovered at sunrise. The temperature in the sun varied from 30 to 45 °C. The dry product was then crushed (Hammer mill, Lister‐DMA) to obtain lafun flour.

### Lafun dough preparation

Lafun dough (1 kg) was prepared using a mixture whose proportions were obtained from preliminary tests with processors: boiled water (631 mL), ogi (158 g) and lafun flour (211 g). Ogi was added to boiling water, which was kept on the fire 30 additional seconds before being removed. Thereafter, lafun flour was added and the mixture was kneaded during 3 min using a kitchen kneader (Nasco‐HM‐990, France).

### Physico‐chemical characterisation

#### Dry matter content and water activity

The dry matter content (DMC) was determined on cassava roots, lafun flour and lafun dough by oven drying at 105 °C to constant weight according to AOAC method (1984). Water activity was determined on lafun flour with a thermo‐hygrometer recorder C056696 (Rotronic Hygrolab 2, Bassersdorf, Switzerland) according to the method described by Anihouvi *et al*. (2006). These analyses were repeated three times.

#### Colour

The colour parameters were determined on cassava roots, lafun flour and lafun dough using a CR 410 chromameter (Chromameter Konica Minolta Optics, INS, Japan, 2012) calibrated with a reference white ceramic whose colour coordinates are *Y* = 86.10; *x* = 0.3194 and *y* = 0.3369. The L*, a*, b* values were recorded, where L* corresponds to the luminance; a* the saturation in red and b* the saturation in yellow. The thickness of the samples was 1.3 cm. The brown index (100‐L*) was calculated as described by Akissoe *et al*. (2001). This analysis was repeated three times.

#### Pasting properties of lafun flour

The pasting properties of the lafun flours were measured using a rheometer (HAAKE, Viscotester iQ‐Air, Dielselstrasse 2, Germany) in accordance with AACC method 61‐02‐01 (2012). A flour‐water suspension of 25 mL with 8% (dry basis) of dry matter was subjected to the following temperature profile, under continuous stirring at 160 rpm: holding at 50 °C for 5 min, heating from 50 °C to 95 °C at 6 °C min^−1^, holding at 95 °C for 5 min, cooling down to 50 °C at 6 °C min^−1^ and then holding at 50 °C for 5 min. Fives parameters were measured: temperature at which starch granules begin to swell and gelatinise and defined as an increase of 0.20 P over a period of 20 s (pasting temperature, PT), time to pasting temperature (pasting time, Pt), peak viscosity (PV), time to peak viscosity (tPV), hot paste viscosity at the end of the plateau at 95 °C (holding strength, HS) and cool paste viscosity raising 50 °C (final viscosity, FV). With them, three additional parameters were calculated: breakdown (PV‐HS), ease of cooking (tPV‐Pt) and setback (FV‐HS). The analyses were performed in duplicate, the mean values were calculated and the viscosity parameters were expressed in Poise (P).

#### Textural analysis of lafun dough

Textural analysis was performed using a texturometer (model TA‐XT plus, Stable Micro Systems, Godalming, UK) equipped with an extrusion cell (OTTAWA cell – A/OTC; extrusion plates A/WBL) with a 50 kg load cell. Three samples of 150 g were collected from the lafun dough obtained from each variety, for a total of forty‐eight samples. Since lafun dough is consumed lukewarm (around 45 °C), the three samples from each variety were wrapped in plastic film and stored in an oven at 45 °C (GFL 3031) for 15 min (0), 5 h (5) and 24 h (24), respectively. The extrusion parameters applied for each sample were extrusion distance of 80 mm and the pre‐test, test and post‐test speeds of 5, 1 and 10 mm sec^−1,^ respectively. Hardness (N) defined as the peak compression force and stickiness (N.sec), which is the negative force area of the compression, were recorded for the lafun dough characterisation. This experimental design was repeated three times.

#### Lafun dough rheology

The rheological behaviour of the lafun dough sample was evaluated using a rheometer (HAAKE Viscotester iQ Air) equipped with parallel planes geometry with a ridged surface to avoid any sliding effect. The measurements were carried out at 25 °C, and the temperature of the sample was controlled by means of a Peltier effect system connected to a refrigerant (ViscothermVT2, Anton Paar GmbH, Graz, Austria). For each variety, samples of 3 × 150 g lafun dough were collected and stored in an oven at 45 °C (GFL 3031) during 15 min (0), 5 h (5) and 24 h (24), respectively. Two sub‐samples of 10 g were collected for each storage duration. Each sub‐sample was placed between the two plates and allowed to stand for 60 s before the measurements, while the excess of dough was removed. Analysis was carried out at a constant frequency of 1 Hz, while strain amplitude varied between 0.001% and 1000%. The storage modulus (G’, a measure of elastic response) and loss modulus (G”, a measure of viscous response) were calculated. The evolution of G' allowed identification of the critical stress τc (Pa) limiting the linear viscoelastic region (LVR). The critical stress was determined when the G’ value decreased more than 5% with respect to the previous average value. This critical stress, corresponding to the LVR G’, was considered as the viscoelastic response. This experimental design was repeated twice.

### Statistical analysis

Data were subjected to descriptive statistics and to analysis of variance (ANOVA), and mean differences compared by the least significant difference (*P* < 0.05). Pearson’s correlations (*P* < 0.05) were determined where appropriate using XLSTAT (Addinsoft, Paris, France, Version 2019).

## Results

### Variability in dry matter content and water activity of cassava varieties and derived lafun

A high variation of dry matter content (DMC) of fresh roots was observed among the varieties tested, ranging from 18.1% to 37.7% (wet basis) (Table 1). The mean DMC in the provitamin A Carotenoid (pVAC) varieties (29.5%) was significantly lower than the mean DMC of local white‐fleshed varieties (35.9%). There was no significant variation in DMC of lafun flour (89.1–91.0%) or of lafun dough (23.6–25.3%) irrespective of the cassava varieties types. The mean water activity of local variety flour samples (*a*_w_ = 0.51) was significantly lower than the mean obtained for samples from both improved white‐fleshed cassava (*a*_w_ = 0.59) and from pVAC varieties (*a*_w_ = 0.60).

**Table 1:** Some indicators of quality of raw cassava root and derived lafun products

Varieties^†^	Types	Cassava roots			Lafun flour				Lafun dough		
		DMC (%)	Colour		DMC (%)	Colour		a_w_	DMC (%)	Colour	
			b	BI		b	BI			b	BI
I011412	Provitamin A carotenoid	22.5 ± 1.0	26.4 ± 0.7	7.3 ± 0.0	89.0 ± 0.4	10.4 ± 0.1	11.0 ± 0.0	0.61 ± 0.01	21.5 ± 0.2	14.6 ± 1.2	13.4 ± 0.0
I011797		30.4 ± 1.1	16.8 ± 0.8	7.9 ± 0.1	88.0 ± 0.2	9.3 ± 0.1	10.0 ± 0.0	0.71 ± 0.00	22.5 ± 0.1	11.4 ± 1.4	14.2 ± 1.8
I070539		18.1 ± 1.1	32.7 ± 2.9	7.6 ± 0.3	90.3 ± 0.7	9.5 ± 0.1	11.2 ± 0.1	0.63 ± 0.01	24.5 ± 1.2	13.8 ± 0.4	18.5 ± 0.7
I070593		29.8 ± 0.3	28.3 ± 2.2	7.8 ± 0.0	85.1 ± 1.6	11.1 ± 0.1	11.1 ± 0.0	0.61 ± 0.01	21.5 ± 0.5	15.0 ± 1.3	14.8 ± 1.0
I083594		34.6 ± 0.7	28.0 ± 2.1	7.6 ± 0.1	91.5 ± 1.2	12.3 ± 0.1	11.1 ± 0.1	0.53 ± 0.01	27.0 ± 0.3	21.2 ± 2.5	9.4 ± 0.9
I083724		36.8 ± 1.3	20.3 ± 0.2	8.0 ± 0.1	90.3 ± 0.5	9.9 ± 0.1	10.4 ± 0.0	0.57 ± 0.01	27.6 ± 0.4	15.1 ± 0.9	15.1 ± 2.1
I090090		34.1 ± 1.4	28.5 ± 1.5	7.4 ± 0.1	89.4 ± 1.0	14.4 ± 0.1	11.0 ± 0.0	0.57 ± 0.01	24.9 ± 0.6	19.8 ± 1.0	13.4 ± 1.5
Mean		29.5^a,*^	25.9^a,*^	7.7^a,1^	89.1^a^	11.0^a,**^	10.8^a,2^	0.60^a^	24.1^a^	15.8^a,***^	14.1^a,3^
Ina‐H (92B/00061)	Improved white‐fleshed	30.9 ± 1.2	3.9 ± 0.2	6.8 ± 0.2	90.4 ± 0.7	6.1 ± 0.0	9.8 ± 0.0	0.58 ± 0.00	23.8 ± 0.3	9.1 ± 0.4	14.4 ± 1.0
MR‐67 (92/0067)		30.1 ± 0.9	3.9 ± 0.1	7.0 ± 0.1	89.3 ± 0.3	6.3 ± 0.1	10.2 ± 0.1	0.64 ± 0.00	23.7 ± 0.2	10.3 ± 0.3	14.1 ± 1.0
Ina Premier (92/0427)		34.0 ± 1.7	4.0 ± 0.0	6.7 ± 0.1	90.1 ± 0.6	6.3 ± 0.2	10.2 ± 0.1	0.63 ± 0.00	26.1 ± 0.4	11.6 ± 0.6	16.4 ± 2.3
Igbèkokpan (92B/00068)		22.6 ± 0.9	4.0 ± 0.0	6.8 ± 0.1	88.1 ± 1.1	6.2 ± 0.0	9.6 ± 0.3	0.52 ± 0.01	26.7 ± 0.0	9.6 ± 0.9	13.2 ± 1.6
Obaïlé (92B/0057)		33.1 ± 1.2	4.0 ± 0.1	6.5 ± 0.2	91.0 ± 0.7	5.9 ± 0.0	9.3 ± 0.1	0.55 ± 0.00	22.9 ± 0.2	10.6 ± 1.2	13.1 ± 2.6
Maniben‐03 (Oko‐Iyawo)		37.7 ± 0.8	4.0 ± 0.0	6.7 ± 0.1	91.4 ± 0.4	6.0 ± 0.0	9.0 ± 0.1	0.56 ± 0.00	24.9 ± 0.5	10.1 ± 1.2	15.7 ± 0.9
RB 89509 (RB or Gbézé)		35.5 ± 0.3	4.0 ± 0.1	6.7 ± 0.1	79.1 ± 1.1	5.6 ± 0.1	9.0 ± 0.1	0.66 ± 0.01	26.3 ± 0.4	8.3 ± 0.4	12.9 ± 0.6
Mean		32.1^a,b^	3.4^b,*^	6.8^b,1^	88.9^a^	6.1^b,**^	9.6^b,2^	0.59^a^	25.3^a^	9.9^b,***^	14.3^a,3^
Ben‐86052 (Ben)	Local white‐fleshed	36.0 ± 0.1	4.0 ± 0.0	6.8 ± 0.2	90.9 ± 0.7	5.4 ± 0.0	9.0 ± 0.1	0.51 ± 0.00	20.9 ± 0.2	8.1 ± 0.3	14.5 ± 0.8
EYEKOFO		35.9 ± 0.6	3.9 ± 0.0	7.1 ± 0.2	91.1 ± 0.5	5.4 ± 0.0	8.9 ± 0.0	0.52 ± 0.00	26.3 ± 0.2	9.7 ± 0.2	13.2 ± 1.9
Mean		35.9^b^	3.9^b,*^	6.9^b,1^	91.0^a^	5.4^b,**^	8.9^c,2^	0.51^b^	23.6^a^	8.9^b,***^	13.8^a,3^

*a*_w_, water activity; BI, Brown index; DMC, Dry matter content in wet basis.

Different letters in the same column indicate significant differences between the three groups of cassava (*P*‐value = 0.05). Different figures at superscript in the same line indicate significant differences of BI between products (cassava roots, lafun flour and lafun dough) (*P*‐value = 0.05). Different letters at superscript in the same line indicate significant differences of b between products (cassava roots, lafun flour and lafun dough) (*P*‐value = 0.05).

^†^ Name of varieties (synonym).

### Change in colour parameters during lafun processing

During processing into lafun, colour of cassava and derivatives was characterised by a yellow index and brown index (Table 1). Both parameters were very similar for local and improved white‐fleshed cassava, but lower than those of the pVAC varieties. The brown index of lafun flour differs significantly between the three types of cassava varieties, the lowest being the local white‐fleshed varieties and the highest the pVAC varieties. For the yellow index of lafun flour, only the pVAC varieties were significantly different, with a reading of 11.0 compared to 6.1 and 5.4, respectively, for the improved and local white‐fleshed varieties. After cooking, no significant difference was observed between the brown indexes of the doughs, but the yellow index showed significant difference between products, with the pVAC varieties having the highest level. The yellow index of pVAC varieties decreased significantly more than 50% during lafun flour production and later increased in lafun dough. For all variety types, the brown index increased significantly both during processing into lafun flour and during cooking into dough.

### Pasting properties of the lafun flours

The pasting properties of lafun flours revealed significant differences between the three types of cassava varieties (Table 2 and Figure S1). Only pasting temperatures, related to the beginning of starch gelatinisation during the heating step, were similar for the three types of cassava varieties, ranging between 68.1 and 69.7 °C. Local white‐fleshed varieties and pVAC had the highest and lowest average peak viscosity values (62.2 and 51.6 P, respectively); however, inside each variety type, large variations were observed. Most varieties reached peak viscosity at similar times (tPV), between 11.8 and 13.6 min, with four notable exceptions: Local white‐fleshed variety Ben‐86052 (Ben) and improved white‐fleshed varieties Ina‐H (92B/00061), Obaïlé (92B/0057) and Maniben‐03 (Oko‐Iyawo) had much higher peak times, between 20.6 and 29.3 min. Such high peak times indicate a gradual increase in viscosity for these varieties, without a distinct viscosity peak in the pasting profile. Breakdown viscosity was highest in pVAC (24.1 P) and lowest in improved white‐fleshed varieties (19.3 P). Final viscosity was significantly highest in local white‐fleshed samples (59.8 P), closely followed by improved white‐fleshed (50.2 P) and then by pVAC varieties (37.5 P). The three types of cassava varieties had significantly different setback values with 19.7, 13.8 and 10.0 P for local white‐fleshed, improved white‐fleshed and pVAC varieties, respectively.

**Table 2:** Pasting properties of lafun flour made from local and improved cassava varieties

Varieties*	Type	Final viscosity FV (P)	Viscosity peak PV (P)	Peak time tPV (min)	Pasting temperature PT (°C)	Breakdown PV‐HS (P)	Setback FV‐HS (P)	Ease of cooking tPV‐Pt (min)
I011412	Provitamin A carotenoid	31.5 ± 0.1	51.4 ± 1.4	11.8 ± 0.2	67.5 ± 2.5	29.0 ± 1.1	9.1 ± 0.2	2.3 ± 0.1
I011797		35.4 ± 0.1	47.6 ± 0.3	12.4 ± 0.0	72.5 ± 0.0	21.5 ± 0.6	9.2 ± 0.4	2.4 ± 0.0
I070539		28.0 ± 0.2	39.3 ± 0.2	12.6 ± 0.1	70.0 ± 0.0	18.0 ± 0.8	6.7 ± 0.4	3.0 ± 0.2
I070593		47.8 ± 0.7	61.2 ± 1.5	12.7 ± 0.1	72.5 ± 0.0	26.2 ± 0.6	12.8 ± 0.2	2.6 ± 0.1
I083594		34.9 ± 0.4	51.1 ± 0.6	12.6 ± 0.2	64.5 ± 0.5	24.9 ± 1.4	8.6 ± 0.4	3.6 ± 0.1
I083724		38.7 ± 0.0	52.5 ± 0.6	12.9 ± 0.2	66.3 ± 1.3	23.4 ± 1.0	9.5 ± 0.4	3.7 ± 0.3
I090090		46.5 ± 0.1	57.9 ± 0.4	12.8 ± 0.0	63.8 ± 0.3	25.5 ± 0.2	14.0 ± 0.3	4.0 ± 00
Mean		37.5^a^	51.6^a^	12.6^a^	68.1^a^	24.1^a^	10.0^a^	3.1^a^
Ina‐H (92B/00061)	Improved white‐fleshed	51.3 ± 0.5	52.1 ± 1.0	20.6 ± 0.7	68.0 ± 0.5	15.3 ± 0.4	14.6 ± 0.1	11.2 ± 0.7
MR‐67 (92/0067)		42.1 ± 0.3	55.8 ± 0.6	12.7 ± 0.7	72.5 ± 0.0	28.8 ± 0.7	15.1 ± 0.4	2.6 ± 0.7
Ina Premier (92/0427)		43.8 ± 0.5	47.1 ± 1.0	13.6 ± 0.1	67.5 ± 0.0	13.4 ± 0.1	10.0 ± 0.4	4.3 ± 0.1
Igbèkokpan (92B/00068)		51.0 ± 0.1	61.5 ± 0.6	13.3 ± 0.1	70.0 ± 0.0	24.0 ± 0.7	13.5 ± 0.1	3.6 ± 0.1
Obaïlé (92B/0057)		52.3 ± 0.2	52.7 ± 0.2	27.7 ± 0.4	68.8 ± 1.3	12.0 ± 0.6	11.6 ± 0.6	18.2 ± 0.6
Maniben‐03 (Oko‐Iyawo)		50.0 ± 2.0	50.3 ± 2.0	28.8 ± 0.3	68.8 ± 1.3	12.8 ± 1.6	12.5 ± 1.6	19.3 ± 0.2
RB 89509 (RB or Gbézé)		61.6 ± 0.7	71.4 ± 0.6	12.9 ± 0.1	72.5 ± 0.0	29.2 ± 0.9	19.3 ± 0.8	2.9 ± 0.1
Mean		50.2^b^	55.8^a,b^	18.5^b^	69.7^a^	19.3^b^	13.8^b^	8.9^b^
Ben‐86052 (Ben)	Local white‐fleshed	64.3 ± 0.3	64.6 ± 0.2	29.3 ± 0.2	72.3 ± 0.3	22.3 ± 0.0	21.9 ± 0.1	19.3 ± 0.2
EYEKOFO		55.4 ± 0.2	59.9 ± 1.3	13.2 ± 0.0	66.3 ± 1.3	22.0 ± 0.8	17.5 ± 0.2	4.0 ± 0.1
Mean		59.8^c^	62.2^b^	21.3^b^	69.3^a^	22.1^a,b^	19.7^c^	11.7^c^

Mean of two replicates ± standard deviation.

* Name of varieties (synonym).

### Hardness and stickiness of lafun dough during storage

**Figure 1:**
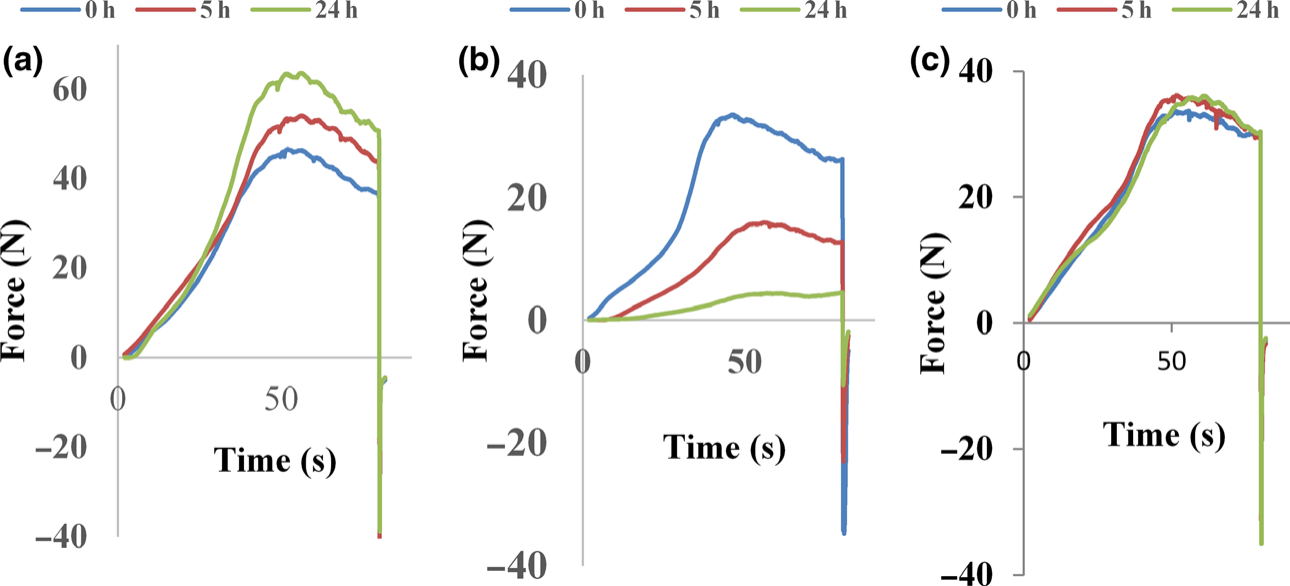
Typical curves of change of lafun dough hardness during storage (0–24 h): (A) Stiffer during storage, (B) Softened during storage and (C) No change of hardness during storage.

Hardness of lafun dough was significantly affected by storage period and cassava variety (Table 3). The pVAC I070539 lafun dough liquefied at the onset of storage period (3.8 N), to the point of becoming unsuitable for the texture and viscoelasticity measurements during storage. Among the remaining 15 lafun doughs studied, three groups of hardness behaviour were observed: increase in hardness, decrease in hardness (softness) and no modification (Fig. 1 and Table 3). Indeed after 5 h of storage, six varieties became stiffer (EYEKOFO, Igbèkokpan (92B/00068), MR‐67 (92/0067), I090090, Ben‐86052 (Ben), and I070593) and two softened (Obaïlè (92B/0057) and I083594). From 5 to 24 h of storage, the six stiffer varieties either continued to harden (EYEKOFO, Igbèkokpan (92B/00068) and MR‐67 (92/0067)) or maintained the level of hardness reached at 5 h. Both softening varieties at 5 h of storage (Obaïlè (92B/0057) and I083594) continued to soften until 24 h, and two more varieties (Ina‐H (92B/00061) and Maniben‐03 (Oko‐Iyawo)) that were initially stable also started to soften until 24 h. The remaining five varieties did not show any change in hardness during the 24 h of storage. No relation was found between hardness behaviour and the varietal type (pVAC, Improved white or Local white); however, both local white varieties belonged to the increasing hardness group (Table 3).

**Table 3:** Change in hardness, stickiness and viscoelastic response (LVR G’) during storage of lafun dough made from local and improved cassava varieties

Varieties*	Type	Hardness (N)				Stickiness (N.sec)			LVR G' (kPa)		
		Storage duration (h)			Behaviour	Storage duration (h)			Storage duration (h)		
		0	5	24		0	5	24	0	5	24
EYEKOFO	Local white	46.6^a^	51.9^b^	59.4^c^	Stiffer during storage	31.5	29.2	29.0	13.1^a^	14.7^a^	12.7^b^
Igbèkokpan (92B/00068)	Improved white	37.5^a^	42.6^b^	50.5^c^		28.2^a^	26.5^a^	18.6^b^	12.4^a^	10.7^b^	11.0^b^
MR‐67 (92/0067)	Improved white	26.7^a^	43.3^b^	59.5^c^		17.9^a^	15.4^b^	15.9^b^	9.7	10.2	8.7
I090090	Improved pVAC	46.6^a^	53.8^b^	53.1^b^		29.6^a^	22.1^b^	16.1^c^	9.3	10.0	9.5
Ben‐86052 (Ben)	Local white	37.3^a^	44.6^b^	45.1^b^		44.9^a^	38.1^b^	29.7^c^	10.8^a^	10.7^a^	8.0^b^
I070593	Improved pVAC	29.7^a^	34.7^b^	36.0^b^		26.9^a^	20.8^b^	15.8^c^	nd	nd	nd
	Mean	37.4	45.2	50.6		29.8	25.4	20.9	11.1	11.3	10.0
	SD	8.3	6.9	9.0		8.8	7.9	6.7	1.7	1.9	1.9
I070539^†^	Improved pVAC	3.8			Softened during storage	14.2			5.6		
Obaïlè (92B/0057)	Improved white	39.1^a^	34.0^b^	24.5^c^		57.7^a^	34.9^b^	24.1^c^	10.3^a^	9.8^a^	6.7^b^
I083594	Improved pVAC	32.7^a^	15.5^b^	7.4^c^		48.6^a^	23.5^b^	14.3^b^	7.1^a^	5.7^a^	5.4^b^
Ina‐H (92B/00061)	Improved white	39.8^a^	40.7^a^	36.0^b^		22.0	18.4	21.1	16.5	15.9	14.2
Maniben‐03 (Oko‐Iyawo)	Improved white	37.2^a^	37.5^a^	31.7^b^		44.4^a^	24.5^b^	14.5^b^	14.4	13.8	10.7
	Mean	26.8	26.9	21.7		32.6	21.8	16.1	9.3	9.4	7.8
	SD	16.3	14.9	13.0		20.2	9.8	6.8	5.6	5.7	4.8
RB 89509 (RB or Gbézé)	Improved white	30.4	31.7	37.5	No change during storage	20.7^a^	16.4^b^	17.2^b^	8.2	8.6	8.4
I011797	Improved pVAC	14.9	17.0	16.2		15.8	13.6	13.4	9.7	10.1	10.3
I083724	Improved pVAC	57.1	61.9	59.4		18.1	17.7	19.8	10.8	10.8	10.9
Ina Premier (92/0427)	Improved white	18.4	19.2	18.0		24.8^a^	15.5^b^	19.6^b^	15.0	16.0	16.0
I011412	Improved pVAC	21.0	22.2	25.6		15.4	13.2	13.9	9.2	10.5	10.4
	Mean	26.4	28.3	28.3		19.2	14.4	15.1	9.7	10.3	10.1
	SD	16.0	17.7	17.6		3.5	2.8	4.9	3.1	3.4	3.7

nd, not determined; pVAC, Provitamin A Carotenoid; SD, standard deviation.

By parameter, in the same line, different letters indicate significant differences while absence of letters indicates no significant difference between storage duration (*P*‐value = 0.05).

* Name of varieties (synonym).

^†^ No data can be collected from 5 h of storage onwards ‘Not detected at the threshold point’.

A significant decrease in stickiness was observed during storage for most of the samples (10 out of 15) with no significant effect of cassava groups (Table 3). In addition, no significant correlation (*r* = 0.38) was observed between hardness and stickiness.

### Viscoelastic properties of lafun dough

For all samples, G’ values are higher than G” values during lafun dough storage (results not shown). The three types of cassava exhibited similar viscoelastic behaviour. However, LVR G’ of 5 out of 14 samples decreased during storage duration (Table 3).

## Discussion

Many indicators, particularly dry matter content (DMC), are often considered in the choice of cassava varieties for specific processing purposes. High DMC in cassava roots is a characteristic preferred by farmers in Benin (Adégbola *et al*., 2013) and in Nigeria (Teeken *et al*., 2018), because it is associated with higher process yield. As reported by several authors (Eleazu & Eleazu, 2012; Esuma *et al*., 2016) in comparative studies between pVAC and white‐fleshed varieties, pVAC cassava included in the current study had lowest DMC in comparison with all white‐fleshed (local and improved) varieties. Accordingly, to enhance adoption of pVAC varieties in the future, breeders will need to focus efforts on increasing DMC of fresh roots. After processing, lafun flour had high DMC (≈90%) and low water activity (mean value ˂0.7) which allows storage at ambient temperature for months, according to Sanni *et al*. (1998) and Pinto *et al*. (2005).

Colour is another important criterion that orients the choice of cassava users for specific purposes. The predominant flesh colour of cassava is white, along with most of its derived products (Vimala *et al*., 2011). However, for pVAC varieties, which have yellow flesh, the colour of the end products must be subject to the consumer acceptance. In this study, two factors were identified that influence colour: Sun‐drying during processing of pVAC into lafun flour decreased significantly yellowness, which might indicate loss in carotenoids as observed by Vimala *et al*. (2011), Maziya‐Dixon *et al*. (2009) and Chavez *et al*. (2007). The increase in brown index in all lafun dough may be due to the Maillard reaction involving utilisation of available reducing sugars or proteins (Akissoe *et al*., 2001; Oyedeji *et al*., 2017).

Regarding the pasting (gelatinisation) properties, our results did not show significant differences among the three types of cassava varieties. Pasting temperatures were close to the range of 66.8–70.4 °C reported by Aryee *et al*. (2006) on cassava flours and lower than those reported by Awoyale *et al*. (2015) on pVAC root starches (76.7 °C) and by Adebayo‐Oyetoro *et al*. (2012) on lafun flour (79.3–83.4 °C). However, this may simply reflect different pasting protocols (e.g. heating rate). Starch composition (amylose/amylopectin ratio) with higher amylose content associated with higher pasting and gelatinisation temperatures (Charles *et al*., 2005). Other studies have found significant variations in amylose content of cassava varieties, in particular Awoyale *et al*. (2015) on three pVAC starches (21.0–24.8%) and Onitilo *et al*. (2007) on 39 improved white‐fleshed starches (19.7–25.1%). The three types of cassava varieties differed significantly according to their peak viscosity. Rosenthal *et al*. (1974) suggested that high peak viscosity contributes to a good texture of paste, which depends on moderately high gel strength. Kaur *et al*. (2007) reported that peak viscosity increases with the starch granule size, which determines the swelling pattern and viscosity profile during pasting. Overall, the local white‐fleshed cassava varieties had high peak viscosity in accordance with the preferences of local processors and consumers for lafun stiff dough. Some of the improved varieties also showed viscosity peaks close to the local white‐fleshed varieties, and even higher in some cases (I070593, I090090, Igbèkokpan (92B/00068) and RB 89509). These improved varieties with high viscosity peaks belong to the variety type with stiffer behaviour during storage (I070593, I090090, Igbèkokpan (92B/00068)) or to the variety type with no significant change during storage (RB 89509). When starch breakdown occurred during the heating process, pVAC flours had higher breakdown. Kaur *et al*. (2007) showed that during the breakdown, the swollen starch granules are disrupted and amylose molecules are continuously leaching out. Eliasson (2004) reported that high breakdown is not desirable since it leads to uneven viscosity and results in the cohesive nature of the starch paste. Hence, in this study, the best lafun paste stability was observed with improved white‐fleshed varieties, which had the lowest breakdown. The three types of cassava varieties also differed significantly according to their final viscosity and setback, indicating different tendencies towards re‐association of amylose molecules upon cooling. Local white‐fleshed varieties in particular had the highest final viscosity and setback. According to Karim *et al*. (2000), the setback refers to the gelling ability of starch which results from the rearrangement of leached amylose molecules. Sanni *et al*. (2001) also reported that higher setback during cooling of lafun indicates higher amylose retrogradation. Tappiban *et al*. (2020) demonstrated that the pasting properties, and in particular the setback, are influenced by the molecular size of amylose and amylopectin. Thus, the larger the amylose and the amylopectin, the higher the value of setback viscosity. Their results showed also that molecular size of amylose and amylopectin is dependent of the cropping environment. Further research is needed to clarify the impact of environmental conditions on the quality of flours and end products. Based on the work reported here, the lower final viscosity and setback observed in lafun from pVAC indicate a weaker network of retrograded amylose in these varieties, possibly reflecting a lower amylose content. In this case and according to Kim *et al*. (1995), these cassava varieties cannot be used for products in which starch stability is required at low temperatures such as adhesives, fillings and products that require refrigeration. Further research should investigate the behaviour of lafun dough at 4 °C, since currently the product is stored at ambient temperature (28–35 °C) only two to three days.

Regardless of flesh colour and variety, the lafun doughs were clustered into three groups according to their hardness behaviour during storage (45 °C for 24 h): either increase, stable or decrease in hardness. In the vast majority of cases, pastes and gels of gelatinised starch tend to increase in hardness during storage, due to starch retrogradation (Karim *et al*., 2000): firstly by amylose retrogradation within minutes after gelatinisation and cooling (Fechner *et al*., 2005), and secondly by amylopectin retrogradation within hours or days depending on storage conditions (Tran *et al*., 2007). In the case of cassava, Rodríguez‐Sandoval *et al*. (2008) reported an increase in hardness for dough produced from cassava parenchyma cooked and stored at either −5 °C or −20 °C during 24 h. In the current study, the decrease in hardness observed in lafun doughs for some varieties was thus unexpected and points to a concurrent phenomenon of retrogradation. One hypothesis is enzymatic activity (amylases) during storage, which resulted in partial hydrolysis of the starch matrix and consequently decrease in hardness. A total of five varieties out of 16 exhibited reduced hardness after storage: Two pVAC (I070539, I083594) and three improved white (Obaïlè (92B/0057), Maniben‐03 (Oko‐Iyawo), Ina‐H (92/00061)). The I070539 variety in particular softened to such an extent that it became liquid‐like and texture measurement was not possible. Interestingly, the three improved white varieties that softened during storage all had high peak times (tPV, Table 2) associated with a gradual increase in viscosity during pasting rather than a distinct viscosity peak. The changes in viscosity observed on the pasting profile reflect the molecular and supramolecular structures of starch and starch granules; therefore, such structures may play a role in the softening during storage of lafun. Softening of lafun dough is an undesirable quality trait, and cassava varieties that behave in this way need to be detected and screened out. The tPV parameter may be relevant for this purpose, given the apparent link between high tPV and the softening during storage of lafun from improved white varieties. This link needs to be confirmed through further study of the influence of cassava variety on the quality of lafun.

In the current study, the hardness of lafun doughs after 24 h storage was associated with higher setback as measured by AACC 61‐02‐01 (2012) on lafun flours, which indicates that amylose may contribute in determining the hardness after storage. However, no significant correlation was identified (*r* = 0.52), which can be explained considering that lafun flour and lafun dough are distinct products: In particular, lafun dough contained added cereal starch (ogi) that may also contribute to increasing the hardness after storage. Frederick (2007) reported that high levels of cereal, in a gluten‐free bread formulation, led to bread with a high hardness, and Onyango *et al*. (2011) showed that the firmness of the crumb of sorghum‐cassava formulations increased when the concentration of cassava starch decreased. When focusing on the group of varieties that exhibit decreasing hardness during storage of lafun dough, the extent of amylose retrogradation, as indicated by the setback, may play a role in limiting the loss of hardness, as shown by the significant correlation (*r* = 0.96) between setback and the rate of decrease in hardness between 0 and 24 h. The differences in textural behaviour among the three groups of cassava varieties may also be due to differences in the damage to starch granules and starch molecules during fermentation, sun‐drying and/or cooking, depending on the cassava variety. Non‐starch polysaccharides present in the lafun, such as cell wall materials, may also influence textural behaviour, either through their quantity or through their mode of degradation caused by hydrolytic enzymes and the lower fermentation pH as reported by Adetunji *et al*. (2016).

A decrease in stickiness was observed during storage for most of the doughs. Addition of maize starch in lafun dough preparation probably affected the stickiness behaviour. As reported by Monthe *et al*. (2019), adhesiveness, which indicates the sticky character of the crumb, is less pronounced for cereal proportions greater than 12.5% and cassava amount lower than 75%, equivalent to a ratio of 1:6 while in our study the ratio is around 1:4 (dry basis). In addition to ingredients, the storage temperature also influenced the stickiness of lafun dough. Indeed, Hadziyev & Steele (1979) reported that a cooling step at 45 °C promoted retrogradation of both extracellular starch and starch within the unruptured cell, thereby decreasing stickiness.

The viscoelastic property is also an indication of lafun dough quality during storage. In comparison to G”, the higher values indicate that lafun doughs experience a lot of mixing and withstand considerable mechanical work (Watanabe *et al*., 1992). Deformations are essentially elastic or recoverable and can be explained by swelling power of starch granules. Shon & Yoo (2006) reported that swelling power and temperatures govern the viscoelastic properties of gelatinised starch dispersion. As reported by Osundahunsi *et al*. (2011), a decrease of viscoelastic response (LVR G’) during storage may be due to the increased stability of the granular integrity caused by strengthening of bonds in the swollen granules of dough due to temperature and storage duration.

## Conclusions

Pasting properties of lafun flour and hardness during lafun dough storage strongly discriminate the 14 improved and two local cassava varieties evaluated in this study, resulting in three textural behaviours during storage: stiffening, softening and no modification. Overall, improved varieties showed poor pasting properties. Among them, only four lafun dough samples (Igbèkokpan (92B/00068), MR‐67 (92/0067), I090090 and I070593) with high peak viscosity had hardness behaviour close to local varieties (EYEKOFO and Ben‐86052 (Ben)). Softening of lafun dough was observed in five improved varieties and likely related to starch hydrolysis during storage, while stiffening was affected by the addition of cereal starch into lafun. Further research needs to be done to clarify the impact of ogi (maize starch) on the texture of lafun, so that appropriate formulations of ogi‐cassava blends can be developed in order to upgrade the use of the improved cassava varieties. Hardness and stickiness change during lafun dough storage, and optimum texture is achieved at around 5 h storage time. The effect of the environmental conditions undergone by the crops and which determine the fine composition of starch and consequently the texture of lafun need to be explored more deeply.

## Author contribution

**Alexandre Bouniol:** Conceptualization (lead); Data curation (lead); Formal analysis (lead); Investigation (equal); Methodology (lead); Software (lead); Writing‐original draft (lead). **Laurent Adinsi:** Conceptualization (lead); Data curation (lead); Formal analysis (lead); Investigation (lead); Methodology (lead); Supervision (equal); Writing‐original draft (equal); Writing‐review & editing (equal). **Segla Wilfrid Padonou:** Conceptualization (equal); Data curation (equal); Methodology (lead); Supervision (lead); Writing‐original draft (lead). **Francis Hotegni:** Data curation (equal); Formal analysis (equal); Investigation (equal). **Désiré Gnanvossou:** Conceptualization (supporting); Resources (supporting); Writing‐review & editing (lead). **Thierry Tran:** Conceptualization (equal); Data curation (supporting); Formal analysis (supporting); Investigation (equal); Methodology (lead); Writing‐review & editing (lead). **Dominique Dufour:** Conceptualization (equal); Funding acquisition (lead); Methodology (equal); Resources (lead); Supervision (lead); Writing‐review & editing (equal). **Joseph Hounhouigan:** Supervision (lead); Validation (lead); Visualization (lead); Writing‐review & editing (lead). **Noël Akissoe:** Conceptualization (lead); Formal analysis (equal); Methodology (lead); Supervision (lead); Writing‐review & editing (lead).

## Conflict of interest

The authors declare that they have no conflict of interest.

## Ethical guidelines

Ethical approval was not required for this research.

### Peer review

The peer review history for this article is available at https://publons.com/publon/10.1111/ijfs.14902.

## Supporting information


**Figure S1.** Discrimination of local white, improved white and provitamin A (pVAC) cassava varieties by principal component analysis (PCA) of the parameters of pasting profiles measurements.

## Data Availability

Research data are not shared.

## References

[ijfs14902-bib-0001] AACC (2012). Methods 61‐02.01 rapid ViscoAnalyzer. In: Approved Methods of the American Association of Cereal Chemist (edited by A. Bridges , 11th edn). Pp. 1–4. St. Paul, MN: Cereals and grains association.

[ijfs14902-bib-0002] Adebayo‐Oyetoro, A.O. , Olatidoye, O.P. , Ogundipe, O.O. , Balogun, I.O. & Apara, T.O. (2012). Effect of local cassava fermentation methods on functional, pasting and sensory properties of *Lafun* . Continental Journal of Agricultural Science, 6, 1–8.

[ijfs14902-bib-0003] Adégbola, Y.P. , Yegbemey, R.N. , Djenontin, I.N.S. *et al*. (2013). Les marchés du manioc et du « gari » dans le Sud et le Centre du Bénin : performances et principales contraintes à leur développement. Cahier d’Agriculture, 22, 293–302.

[ijfs14902-bib-0004] Adegunwa, M.O. , Sanni, L.O. & Maziya‐Dixon, B. (2012). Effects of fermentation length and varieties on the pasting properties of sour cassava starch. African Journal of Biotechnology, 10, 8428–8433.

[ijfs14902-bib-0005] Adetunji, I.A. , Clou, H. , Walford, N.S. & Taylor, N.R.J. (2016). Complementary effects of cell wall degrading enzymes together with lactic acid fermentation on cassava tuber cell wall breakdown. Industrial Crops and Products, 90, 110–117.

[ijfs14902-bib-0006] Akissoe, N.H. , Hounhouigan, J.D. , Bricas, N. , Vernier, P. , Nago, M.C. & Olorunda, O.A. (2001). Physical, chemical and sensory evaluation of dried yam (*Dioscorea rotundata*) tubers, flour and amala, a flour‐drived product. Tropical Science, 41, 151–155.

[ijfs14902-bib-0007] Anggraini, V. , Sudarmonowati, E. , Hartati, N.S. , Suurs, L. & Visser, R.G.F. (2009). Characterization of cassava starch attributes of different genotypes. Starch‐Stärke, 61, 472–481.

[ijfs14902-bib-0008] Anihouvi, V.B. , Ayernor, G.S. , Hounhouigan, J.D. & Sakyi‐Dawson, E. (2006). Quality characteristics of lanhouin: a traditionally processed fermented fish product in the Republic of Benin. African Journal of Food and Agriculture Nutrition and Development, 6, 1–15.

[ijfs14902-bib-0009] AOAC . (1984). Official Methods of Analysis. Association of Official Analytical Chemists, 14th ed. Arlington: AOAC.

[ijfs14902-bib-0010] Aryee, F.N.A. , Oduro, I. , Ellis, W.O. & Afuakwa, J.J. (2006). The physicochemical properties of flour samples from the roots of 31 varieties of cassava. Food Control, 17, 916–922.

[ijfs14902-bib-0011] Awoyale, W. , Sanni, L.O. , Shittu, T.A. & Adegunwa, M.O. (2015). Effect of varieties on the functional and pasting properties of biofortified cassava root starches. Journal of Food Measurement and Characterization, 9, 225–232.

[ijfs14902-bib-0012] Ayetigbo, O. , Latif, S. , Abass, A. & Müller, J. (2018). Comparing characteristics of root, flour and starch of biofortified yellow‐flesh and white‐flesh cassava variants, and sustainability considerations: a review. Sustainability, 10, 3089–3120.

[ijfs14902-bib-0013] Charles, A.L. , Chang, Y.H. , Ko, W.C. , Sriroth, K. & Huang, T.C. (2005). Influence of amylopectin structure and amylose content on the gelling properties of five cultivars of cassava starches. Journal of Agricultural and Food Chemistry, 53, 2717–2725. 15796616 10.1021/jf048376+

[ijfs14902-bib-0014] Chavez, A.L. , Sanchez, T. , Ceballos, H. *et al*. (2007). Retention of carotenoids in cassava roots submitted to different processing methods. Journal of the Science of Food Agriculture, 87, 388–393.

[ijfs14902-bib-0015] El Sharkawy, M. (2004). Cassava biology and physiology. Plant Molecular Biology, 56, 481–501. 15669146 10.1007/s11103-005-2270-7

[ijfs14902-bib-0016] Eleazu, C.O. & Eleazu, C.C. (2012). Determination of the proximate composition, total carotenoid, reducing sugars and residual cyanide levels of flours of 6 new yellow and white cassava varieties. American Journal of Food Technology, 7, 642–649.

[ijfs14902-bib-0017] Eliasson, A.C. (2004). Starch in Food: Structure, Function and Applications. New York, NY: Woodhead/CRC Press.

[ijfs14902-bib-0018] Esuma, W. , Kawuki, R.S. , Herselman, L. & Labuschagne, M.L. (2016). Diallel analysis of provitamin A carotenoid and dry matter content in cassava (*Manihot esculenta* Crantz). Breeding Science, 66, 627–635. 27795688 10.1270/jsbbs.15159 PMC5010302

[ijfs14902-bib-0019] Fechner, P.M. , Wartewig, S. , Kleinebudde, P. & Neubert, R.H.H. (2005). Studies of the retrogradation process for various starch gels using Raman spectroscopy. Carbohydrate Research, 340, 2563–2568. 16168973 10.1016/j.carres.2005.08.018

[ijfs14902-bib-0020] Frederick, E.J. (2007). Effect of sorghum flour composition and particle size on quality of gluten‐free bread. Thesis, 123 p. 10.1177/1082013214523632 24519987

[ijfs14902-bib-0021] Hadziyev, D. & Steele, L. (1979). Dehydrated mashed potatoes – chemical and biochemical aspects. Advances in Food Research, 25, 55–136.

[ijfs14902-bib-0023] Karim, A.A. , Norziah, M.H. & Seow, C.C. (2000). Methods for the study of starch retrogradation. Food Chemistry, 71, 9–36.

[ijfs14902-bib-0024] Kaur, L. , Singh, J. , McCarthy, O.J. & Singh, H. (2007). Physico‐chemical, rheological and structural properties of fractionated potato starches. Journal of Food Engineering, 82, 383–394.

[ijfs14902-bib-0025] Kim, Y.S. , Wiesenborg, D.P. , Orr, P.H. & Grant, L.A. (1995). Screening potato starch for novel properties using differential scanning colorimeter. Journal of Food Science, 60, 1060–1065.

[ijfs14902-bib-0026] Maziya‐Dixon, B. , Dixon, A.G.O. & Ssemakula, G. (2009). Changes in total carotenoid content at different stages of traditional processing of yellow‐fleshed cassava genotypes. International Journal of Food Science and Technology, 44, 2350–2357.

[ijfs14902-bib-0027] Monthe, O.C. , Grosmaire, L. , Nguimbou, R.C. *et al*. (2019). Rheological and textural properties of gluten‐free doughsand breads based on fermented cassava, sweet potato and sorghum mixed flours. LWT – Food Science and Technology, 101, 575–582.

[ijfs14902-bib-0028] Nago, M. , Tétégan, E. , Matencio, F. & Mestres, C. (1998). Effects of maize type and fermentation conditions on the quality of Beninese traditional ogi, a fermented maize slurry. Journal of Cereal Science, 28, 215–222.

[ijfs14902-bib-0029] Ojewumi, M.E. , Emetere, M.E. , Ogboso, O.A. & Bolujo, E.O. (2019). Rheological properties of cassava and corn starch. *International* . Research Journal of Applied Sciences, Engineering and Technology, 5, 11.

[ijfs14902-bib-0030] Omodamiro, R.M. , Iwe, M.O. & Ukpabi, U.J. (2007). Pasting and functional properties of *lafun* and starch processed from some improved cassava genotypes in Nigeria. Nigerian Institute of Food Science and Technology, 25, 122–129.

[ijfs14902-bib-0031] Onitilo, M.O. , Sanni, L.O. , Oyewole, O.B. & Maziya‐Dixon, B. (2007). Physicochemical and functional properties of sour starches from different cassava varieties. International Journal of Food Properties, 10, 607–620.

[ijfs14902-bib-0032] Onyango, C. , Mutungi, C. , Unbehend, G. & Lindhauer, M.G. (2011). Rheological and textural properties of sorghum‐based formulations modified with variable amounts of native or pregelatinised cassava starch. LWT‐Food Science and Technology, 44, 687–693.

[ijfs14902-bib-0033] Osundahunsi, F.O. , Seidu, T.K. & Mueller, R. (2011). Dynamic rheological and physicochemical properties of annealed starches from two cultivars of cassava. Carbohydrate Polymers, 83, 1916–1921.

[ijfs14902-bib-0034] Oyedeji, A.B. , Sobukola, O.P. , Henshaw, F. *et al*. (2017). Effect of frying treatments on texture and colour parameters of deep fat fried yellow fleshed cassava chips. Journal of Food Quality, 2017, 1–10.

[ijfs14902-bib-0035] Pinto, D.M. , Lajolo, M.F. & Genovese, M.I. (2005). Effect of storage temperature and water activity on the content and profile of soflavones, antioxidant activity, and in vitro protein digestibility of soy protein isolates and defatted soy flours. Journal of Agricultural and Food Chemistry, 53, 6340–6346. 16076116 10.1021/jf0502451

[ijfs14902-bib-0036] Rodríguez‐Sandoval, E. , Fernández‐Quintero, A. , Sandoval‐Aldana, A. & Quicazán, M.C. (2008). Effect of cooking time and storage temperature on the textural properties of cassava dough. Journal of Texture Studies, 39, 68–82.

[ijfs14902-bib-0037] Rosenthal, F.R.T. , Nakamura, T. , Espindola, A.M.C. & Jochimek, M.R. (1974). Structure of starch granules. Die Starke, 26, 50–55.

[ijfs14902-bib-0038] Sanni, L.O. , Ikuomola, D.P. & Sanni, S.A. (2001). Effect of length of fermentation and varieties on the qualities of sweet potato gari processing. *8th triennial symposium of the international society for tropical root crops IITA Ibadan, Nigeria*. Pp. 208–211.

[ijfs14902-bib-0039] Sanni, L.O. , Oyewole, O.B. & Olowogbade, D.V. (1998). Effect of drying methods on lafun (fermented cassava flour). Tropical Sciences, 38, 1–4.

[ijfs14902-bib-0040] Shon, J.K. & Yoo, B. (2006). Effect of acetylation on rheological properties of rice starch. Starch, 58, 177–185.

[ijfs14902-bib-0041] Tappiban, P. , Sraphet, S. , Srisawad, N. *et al*. (2020). Effects of cassava variety and growth location on starch fine structure and physicochemical properties. Food Hydrocolloids, 108, 1–11.

[ijfs14902-bib-0042] Teeken, B. , Olaosebikan, O. , Haleegoah, J. *et al*. (2018). Cassava trait preferences of men and women farmers in Nigeria: implications for breeding. Economic Botany, 72, 263–277. 30573920 10.1007/s12231-018-9421-7 PMC6267705

[ijfs14902-bib-0043] Tran, T. , Piyachomkwan, K. & Sriroth, K. (2007). Gelatinization and thermal properties of modified cassava starches. Starch/Stärke, 59, 46–55.

[ijfs14902-bib-0044] Vimala, B. , Thushara, R. , Nambisan, B. & Sreekumar, J. (2011). Effect of processing on the retention of carotenoids in yellow‐fleshed cassava (*Manihot esculenta* Crantz) roots. International Journal of Food Science and Technology, 46, 166–169.

[ijfs14902-bib-0045] Watanabe, E. , Bell, A. & Brockway, B. (1992). Rheological studies on wheat flour dough. I. Empirical and fundamental methods. Food Control, 3, 97–101.

