# Supplementary material for: Rheological and textural properties of lafun, a stiff dough, from improved cassava varieties
Source: Int J Food Sci Technol. 2020 Nov 28;56(3):1278–88. doi: 10.1111/ijfs.14902 (PMC7984036; doi:10.1111/ijfs.14902)

**Supplementary Material**

**Rheological and textural properties of lafun, a stiff dough, from improved cassava varieties**

Alexandre Bouniol^1,2&4^, Laurent Adinsi^1*^, Sègla Wilfrid Padonou^1&5^, Francis Hotegni^1^, Désiré Gnanvossou^6^, Thierry Tran^4&7^, Dominique Dufour^3^, Djidjoho Joseph Hounhouigan^1^, Noël Akissoé^1^

^1^ Laboratoire de Sciences des Aliments, Faculté des Sciences Agronomiques, Université d’Abomey-Calavi, 03 BP 2819 Jéricho, Benin

^2^ CIRAD, UMR QUALISUD, Cotonou, Benin

^3^ CIRAD, UMR QUALISUD, F-34398 Montpellier, France

^4^ Qualisud, Univ Montpellier, CIRAD, Montpellier SupAgro, Univ d’Avignon, Univ de La Réunion, Montpellier, France.

^5^ ESTCTPA, Université Nationale d’Agriculture, 01 BP 55 Porto-Novo, Bénin

^6^ International Institute of Tropical Agriculture (IITA), 08 BP 0932 Tri Postal, Cotonou, Bénin

^7^ The Alliance of Bioversity International and the International Center for Tropical Agriculture (CIAT), CGIAR Research Program on Roots Tubers and Bananas (RTB), Apartado Aéreo 6713, Cali, Colombia

*Corresponding author: adinsil2003@yahoo.fr

Supplementary Figure S1: Discrimination of Local white, Improved white and provitamin A (pVAC) cassava varieties by principal component analysis (PCA) of the parameters of pasting profiles measurements.


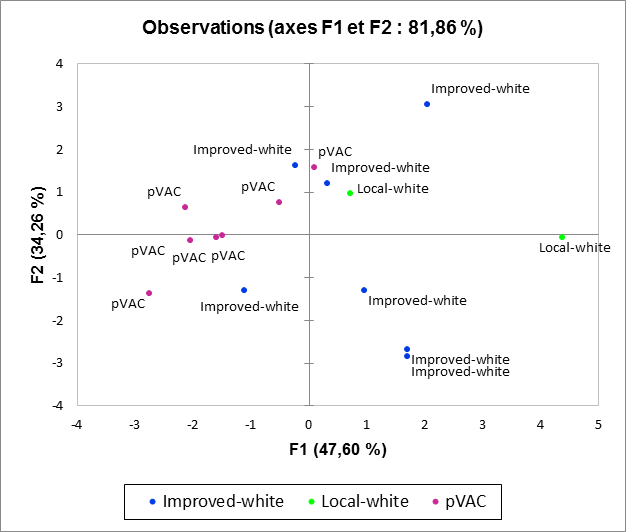


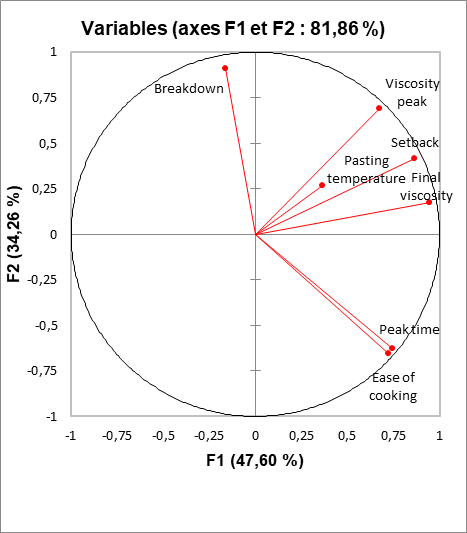

Supplement: Supplementary file 1 — Figure S1. Discrimination of local white, improved white and provitamin A (pVAC) cassava varieties by principal component analysis (PCA) of the parameters of pasting profiles measurements. [file IJFS-56-1278-s001.docx]
